# Preparation of a PM_2.5_-like reference material in sufficient quantities for accurate monitoring of anions and cations in fine atmospheric dust

**DOI:** 10.1007/s00216-017-0670-6

**Published:** 2017-10-02

**Authors:** Jean Charoud-Got, Giovanni Emma, John Seghers, Marie-France Tumba-Tshilumba, Anna Santoro, Andrea Held, James Snell, Håkan Emteborg

**Affiliations:** European Commission, Joint Research Centre, Retieseweg 111, 2440 Geel, Belgium

**Keywords:** PM_2.5_, Reference material, Anions, Cations, Freeze drying, Air quality

## Abstract

A reference material of a PM_2.5_-like atmospheric dust material has been prepared using a newly developed method. It is intended to certify values for the mass fraction of SO_4_^2−^, NO_3_^−^, Cl^−^ (anions) and Na^+^, K^+^, NH_4_^+^, Ca^2+^, Mg^2+^ (cations) in this material. A successful route for the preparation of the candidate reference material is described alongside with two alternative approaches that were abandoned. First, a PM_10_-like suspension was allowed to stand for 72 h. Next, 90% of the volume was siphoned off. The suspension was spiked with appropriate levels of the desired ions just prior to drop-wise shock-freezing in liquid nitrogen. Finally, freeze drying of the resulting ice kernels took place. In using this approach, it was possible to produce about 500 g of PM_2.5_-like material with appropriate characteristics. Fine dust in 150-mg portions was filled into vials under an inert atmosphere. The final candidate material approaches the EN12341 standard of a PM_2.5_-material containing the ions mentioned in Directive 2008/50/EC of the European Union. The material should be analysed using the CEN/TR 16269:2011 method for anions and cations in PM_2.5_ collected on filters. The method described here is a relatively rapid means to obtain large quantities of PM_2.5_. With access to smaller freeze dryers, still 5 to 10 g per freeze-drying cycle can be obtained. Access to such quantities of PM_2.5_-like material could potentially be used for different kinds of experiments when performing research in this field.

**Graphical abstract:**
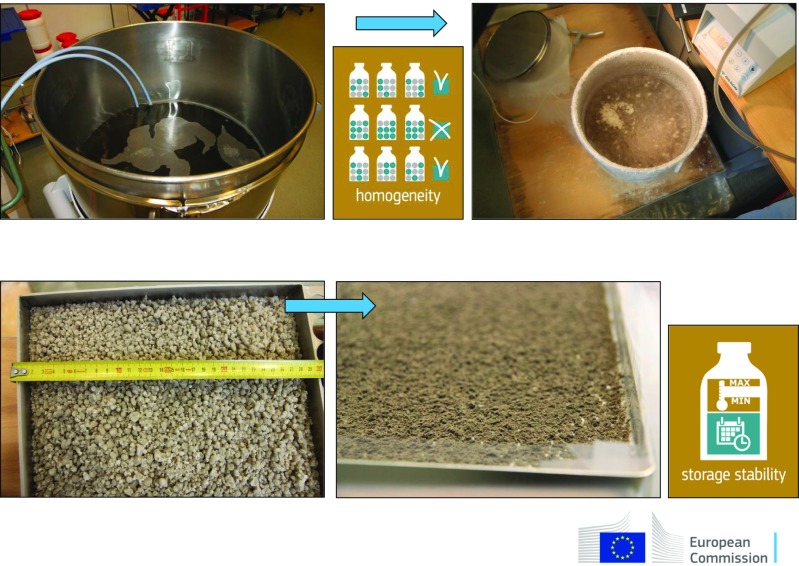
The novelty of the method lies in transformation of a suspension with fine particulate matter to a homogeneous and stable powder with characteristics similar to air-sampled PM_2,5_. The high material yield in a relatively short time is a distinct advantage in comparison with collection of air-sampled PM_2,5_

The novelty of the method lies in transformation of a suspension with fine particulate matter to a homogeneous and stable powder with characteristics similar to air-sampled PM_2,5_. The high material yield in a relatively short time is a distinct advantage in comparison with collection of air-sampled PM_2,5_

## Introduction

The contamination of ambient air by airborne dust, so-called PM_10_ and PM_2.5_, is a major problem in many densely populated parts of the world [1]. In a very comprehensive review article, Fuzzi et al. [2] describe many of the main characteristics of this kind of pollution, ranging from particle sources, composition and effects on human health and climate. The primary sources are traffic, industry, domestic fuel burning and natural sources from volcanos, soil dust and sea spray particles. Also, there are unspecified sources [2]. These particles are known to cause premature deaths and respiratory problems among the general population, especially in heavily contaminated areas [3]. In this context, the PM_2.5_ particles are notoriously dangerous as they can penetrate deep into the lungs and may even pass into the bloodstream [3, 4]. Since atmospheric pollution is not restricted to the borders of any particular country, transnational legislation is ultimately required in addressing this issue. In the European Union, Directive 2008/50/EC has been enforced to monitor and reduce the contamination levels of PM_10_ and PM_2.5_ particles [5]. The legislation also addresses the need to assess natural background levels of particles in rural areas for subtraction from the total particle load. The reference material described in this work specifically addresses the latter aspect.

As a result, quantification of the mass of airborne dust suspended in ambient air (in μg/m^3^) is necessary following Directive 2008/50/EC and applying the definition of particulate matter given in EN12341 [6]. According to this definition, PM_10_ and PM_2.5_ are particulate matter that passes through a size-selective inlet for the sampling and measurement of PM_10_ and PM_2.5_, with a 50% efficiency cut-off at 10 and 2.5 μm aerodynamic diameter, respectively. Since such particles originate from both anthropogenic and natural sources, it is required to measure anions SO_4_^2−^, NO_3_^−^, Cl^−^ and cations Na^+^, K^+^, NH_4_^+^, Ca^2+^, Mg^2+^ in PM_2.5_ collected at rural background locations. The main purpose of measuring these ions is to make adequate information available on natural background levels to be subtracted from enhanced levels in more polluted areas. The ambient air standard gravimetric measurement method, as given in EN 12341 from 2014, must be used for the collection and quantification of PM_10_ and PM_2.5_ [6]. CEN/TR 16269:2011 has been made available to assist the laboratories performing measurements of anions and cations in PM_2.5_ using ion chromatography [7]. Furthermore, Directive 2008/50/EC requires the measurement laboratories to adhere to ISO/IEC 17025 for traceability, quality assurance and mutual recognition of the measurement results [8]. A pivotal means of ensuring accurate and traceable measurement results is to analyse an appropriate certified reference material (CRM) alongside with routine samples. For PM_10_-like materials, two CRMs already exist since 2010, which are available from the EC-JRC (European Commission—Joint Research Centre, Geel, Belgium) in support of Directive 2004/107/EC [9]. To this end, one material (ERM-CZ100) is certified for selected polycyclic aromatic hydrocarbons and the other (ERM-CZ120) is certified for arsenic, cadmium, mercury and nickel [10, 11]. The approach adopted for the preparation of these materials was based on jet-milling of dust collected from a Warsaw road tunnel. Since the material obtained approached the definition of the PM_10_-material characteristics, it was named “PM_10_-like” material. Sufficient amounts of the PM_10_-like material were retained with future projects in mind and were therefore selected as the starting material for the preparation of the PM_2.5_-like material. Additional information about the PM_10_ starting material can be found in the individual certification reports [10, 11].

The main purpose of the work described here was to prepare a sufficient quantity of a PM_2.5_-like material containing the ions SO_4_^2−^, NO_3_^−^, Cl^−^, Na^+^, K^+^, NH_4_^+^, Ca^2+^ and Mg^2+^ common to particles of natural origin. The major challenge was to produce a sufficient amount of material that approached the EN12341 definition of PM_2.5_, which was homogeneous enough with respect to these major ions [6, 12]. In addition, it had to be possible to handle the reference material in a laboratory atmosphere without significant uptake of water while maintaining physico-chemical material properties and a similar analytical/extraction behaviour as PM_2.5_ material collected on filters from ambient air.

To illustrate the impracticality of direct sampling from air, one has to sample 20 million m^3^ of air, containing 30 μg/m^3^ of PM_2.5_ using one standard filter-sampler with a 50% collection efficiency to obtain 300 g of material. Since the standard filter-sampler type has a nominal flow rate of 2.3 m^3^ per hour, it translates into almost 1000 years of run-time for sampling. Even with high volume samplers which operate at 30 m^3^/h, one would need 76 years to collect these 300 g. Numerous practical limitations obviously apply to such long sampling times [6]. Potentially, a battery for example fifty 30 m^3^/h samplers could be used each of them having a set of exchangeable filters. Such an approach would not be without technical challenges either especially since the standard method employs a nominal sampling period of 24 h. In such a case, the collection time would drop to 1.5 years run-time. Alternative approaches had therefore to be developed to produce this reference material.

## Materials and methods

### PM_10_-like starting material

As already mentioned, the starting material was dust collected from the road tunnel Wisłostrada in Warsaw, Poland [10, 11]. First, the material was sieved on a 500-μm stainless steel mesh and subsequently on a 250-μm nylon mesh. After that, it was milled using an Alpine Jet mill (Alpine 50ZPS, Augsburg, DE) employing a ceramic classifier wheel which allows control of the top particle size that can leave the milling system. About 12 kg of PM_10_-like dust was produced. Subsequently, the dust was homogenised using a DYNA-MIX® CM 200 three-dimensional mixer (WAB, Basel, CH). The particle size distribution was measured by laser diffraction (Sympatec Helos, Clausthal, DE) and resulted in a top particle size of about 20 μm, whereas 50% of the cumulative volume distribution was below 8 μm approaching the EN1234 definition of a PM_10_ material. A separate portion of 4.6 kg PM_10_ material was stored at −20 °C to be used as the starting material for a PM_2.5_-like dust material.

Three approaches were tested for the further reduction of particle size of the PM_10_-like material. One was based on a dry route (jet-milling), and two approaches were based on a wet route where a suspension containing tiny particles of the starting material was made. After that, the water from these suspensions was removed either by spray drying or by freeze drying following sedimentation.

### Jet-milling of PM_10_-like material

A jet mill grinds materials by using a high-speed jet of compressed air to impact particles onto each other. The equipment used for the experiment was a Hosokawa Alpine Picoline (Alpine, Augsburg, DE), which is a bench-top model with a stainless steel classifier wheel of 20 mm diameter with a maximum speed of 60,000 rpm. Similar to the larger process jet mill used for preparing the PM_10_-like material, the speed of the classifier wheel is an important parameter for controlling the top particle size. Similarly, the feeding speed of material into the fluidised bed of the jet mill is also important for achieving efficient milling. The milled powder was collected through a Teflon-coated filter in a polyethylene plastic container. The air was extracted from the grinding chamber through the filter by a vacuum cleaner connected with an adjustable valve. The compressed air pressure applied was 6 bar.

### Sedimentation—separating larger particles from small

A 10-L glass bottle was filled with 10 L of Type 2 water from a Merck Millipore system (Billerica, MA, USA); next, 8 g of Triton® X-100 (Merck, Darmstadt, DE) was added. The contents were thoroughly mixed through manual shaking. After that, 70 g of PM_10_ powder was added, and the bottle was vigorously shaken again. Subsequently, the bottle was placed in an ultrasonic bath for 6 h at 37 kHz, 100% power, in sweep mode (Elmasonic P, Elma Schmidbauer, Singen, DE). The temperature at the end of the ultra-sonication was 53–58 °C. Finally, the contents of two of such 10 L bottles were pooled in a 20 L glass bottle and left standing for at least 72 h at room temperature. After sedimentation of the coarser particles, the suspension with the finer particles was transferred by siphoning 90% of the volume into a polyethylene plastic drum with a screw-cap lid. Care was taken to avoid turbulence in the precipitated layer during transfer to prevent larger particles from being released back into the suspension. A sample was taken at the end of the siphoning to confirm the absence of large particles by measuring the particle size distribution. Filled drums were weighed and stored at 4 °C until further use.

Before further manipulation, the suspension was spiked with a mixture of nitrate salts to obtain a final dry powder with levels of ions from 1.5 to 70 g/kg. These levels were considered high enough for eventually assigning certified values and low enough to be representative of samples collected on filters from rural areas. Spiking was performed using a stock solution prepared by mixing 3 g of KNO_3_, 8 g of Mg(NO_3_)_2_ 6 H_2_O and 2 g of NH_4_NO_3_ (Sigma-Aldrich, St. Louis, MO, USA) in 100 mL Type 1 water. This recipe corresponded to a concentration of 11.6 g/L of K^+^, 4.5 g/L of NH_4_^+^, 72.6 g/L NO_3_^−^ and 7.6 g/L of Mg^2+^. This stock solution was used to spike all suspensions containing fine particles. For every 15 kg of PM_2.5_ suspension, 4.0 g of the stock solution was added. Levels of SO_4_^2−^, Cl^−^, Na^+^ and Ca^2+^ were high enough in the starting material and were not increased by spiking. Just after addition of the spiking solution, the plastic drum containing the suspension was placed for 30 min in the ultrasonic bath under continuous stirring with a Teflon paddle.

### Transformation of the suspension into a powder

- Spray drying

A mini spray dryer B-290 (Büchi, Essen, DE) was used to dry the suspension following apparently successful initial tests. The equipment employed a maximal air flow of 35 m^3^/h of air heated to a maximum temperature of 220 °C. The nominal drying capacity was 1 L water/h delivered by a peristaltic pump with adjustable speed. The suspension containing the PM_2.5_ particles was nebulised through a nozzle of 0.7 mm aperture using nitrogen as nebuliser gas with a variable flow from 200 to 800 L/h. The dried powder was collected in a small glass container (100 mL) attached to the glass cyclone placed after the drying chamber. The cyclone and the collection container were covered by thick thermal insulation to avoid collection of a wet pasty product on the colder parts of the spray dryer. The suspension was continuously stirred by a Teflon paddle mounted on an electrical motor set at 50 rpm during the spray drying experiments.

(b) Freeze drying following shock-freezing

Freeze drying is a gentle drying technique where water removal occurs at low temperature and low pressure. The water is evaporated directly from solid ice into vapour which is then trapped on a much colder condenser surface. Gradually, the water is removed by slowly increasing the temperature in the system, and the resulting material is dried. As with all freeze-drying processes, the material first has to be frozen. The suspension contained about 1.8 g fine particles/kg, but regular freezing in the freeze dryer was too slow and resulted in concentrated bands of particles in the ice-matrix, which led to a poor end-product.

In avoiding coalescence phenomena during freezing, the suspension was submitted to drop-wise shock-freezing, i.e. nearly instant freezing in liquid nitrogen. A Gilson peristaltic pump (Gilson, Villiers-le-Bel, FR) with four silicone tubes of 8 mm o.d. and 4 mm i.d. were used. The suspension was pumped from the plastic drum containing 15 kg of spiked suspension at a speed of around 1.5 kg/h per tube, resulting in a freezing capacity of 6 kg/h. The pump speed was set so that the suspension could allow for single droplets to be formed at the outlet of the tubes. The drops subsequently fell in liquid nitrogen contained in four stainless steel containers (one tube per container). The drops that fell into the liquid nitrogen caused it to boil, resulting in vigorous turbulence leading to the formation of single ice kernels of 5 to 7 mm diameter. Every 20 min, liquid nitrogen was topped up to maintain a sufficient volume in the containers. The pump and the plastic drum containing suspension were placed on an analogue orbital shaker to keep the small particles suspended (VWR, Standard 5000 shaker, Leuven, BE). Also, a Teflon paddle mounted on the separate stand was immersed in the suspension for mixing. Every 40 min, the ice kernels were collected by pouring the liquid nitrogen over a stainless steel colander equipped with 3 mm diameter holes. The liquid nitrogen was collected in a second recipient and recycled while the ice kernels were placed in a plastic crate, which was then stored at −70 °C. The collection was rapid since all four containers with ice kernels and liquid nitrogen could be harvested within 5 min. The suspension was shock-frozen on the day of spiking with nitrate salts to avoid losses of nitrate due to bacterial activity.

Portions of ice kernels were scooped up from the plastic crate taken from the −70 °C freezer and spread out on flat-bottom metallic freeze-drying trays. The trays were covered with a polyethylene plastic foil to facilitate material transfer after drying. Before filling, the trays had been kept at −20 °C for at least 12 h to avoid melting of the ice kernels in contact with the trays if kept at ambient temperature. Once filled with 1.25-kg portions of ice kernels, the trays were immediately placed on temperature-controlled shelves inside the freeze dryer. The shelves were kept at −25 °C during the loading phase under a constant flow of nitrogen. The freeze dryer was an Epsilon 2-100D model equipped with a Plexiglass loading door. This system used has a 100 kg nominal ice condenser capacity (Martin Christ, Osterode, DE). In total, 271.5 kg of shock-frozen suspension in the form of ice kernels was loaded in the freeze dryer and dried by applying five freeze-drying cycles (4 × 58.5 kg + 37.5 kg for the last run). The total duration of one cycle was about 90 h using the program given in Table 1. After completing the drying cycle, the freeze dryer was flushed with nitrogen before opening the main door of the drying chamber and opening of the narrow mailbox like Plexiglas loading door inside. On each tray, approximately 2 g of light-grey fine dust was remaining. One by one, the trays were emptied by rapidly transferring the plastic foil into a glove box, constantly flushed with dry nitrogen gas. Inside the glove box, the plastic foils were emptied in a drum made of stainless steel to avoid build-ups of static electricity. In this manner, the fine dust from five freeze-drying cycles was transferred to the glove box and filled directly batch by batch.

**Table 1:** Freeze drying program used to dry the shock-frozen suspension containing particulate matter

Step	Freeze-drying phase	Duration hh:mm	Shelf temperature, °C	Pressure, mbar
1	Loading	00:20	−25	A
2	Freezing	01:00	−20	A
3	Sublimation	14:00	−20	0.2
4	Sublimation	24:00	−10	0.2
5	Sublimation	51:00	−5	0.2
6	Secondary drying	03:00	20	0.05

### Filling of PM_2.5_ like dust in vials

The filling of the dry powder was performed in a glove box under constant flushing with dry nitrogen since the material is hygroscopic. A filling machine was placed in the glove box with all the vials, Teflon-coated inserts, aluminium crimp caps and relevant tools for the filling and capping of the vials. The filling device was a unidirectional asymmetric vibrating feeder model FD-SPAc 4A (MCPI, Meythet, FR). The machine parts in contact with the PM_2.5_ material were coated with diamond-like carbon (DLC). Vials were filled with a target mass of 165 mg to ensure a minimum mass of 150 mg in every vial. An antistatic ion blower was used to remove electrical charges during weighing in the very dry environment. Only small quantities of the powder were loaded into the hopper progressively to avoid the formation of clogged material that could have had an adverse influence on the precision of the mass filled and possibly on the composition. Once filled, the vials were immediately closed with a Teflon-coated rubber insert. The insert was manually secured with an aluminium crimp cap. The temperature and relative humidity inside the glove box were recorded every 2 min using a data logger. The relative humidity in the glove box never exceeded 5% RH during the filling sequence. In total, 2843 vials were filled in this way.

No attempt was made to homogenise the entire powder bulk and thereafter proceeding to filling which is normal practice when processing reference materials. The mixing was not attempted because of the risk of irreversibly destroying the material. After labelling according to fill-order, the vials were placed one by one in aluminised sachets and were then thermally sealed for additional protection of the material (Daklapack, Lelystadt, NL). Records were taken to be able to distinguish between different freeze-drying batches using the sequential fill-order number associated with each vial. The homogeneity test performed after filling dictates the batches that could constitute the reference material stock since it was anticipated that the procedure was repeatable enough to prevent excessive between-batch variation.

### Ion chromatography

Ion chromatography was performed in accordance with CEN/TR 16269:2011. The extraction time was 30 min for exhaustive extraction of the ions as given in the standard. The ion chromatography system was an 850 Professional IC system from Metrohm with a 942 extension module Vario (Herisau, CH). For anion and cation separation, a Metrosep A Supp 5—250/4.0 mm column with 0.7 mL/min flow rate of a solution 3.2 mM Na_2_CO_3_/1.0 mM NaHCO_3_ and Metrosep C6—150/4.0 mm column with 0.9 mL/min flowrate of a solution 1.7 mM dipicolinic acid/1.7 mM HNO_3_ were used. Both columns were installed together with a Metrosep A Supp 4/5 Guard/4.0 guard column. Detection was achieved by conductivity using a suppressor column.

### Particle size analysis and Karl Fischer titration for the determination of residual water content

The particle size distribution was determined by laser diffraction using a Helos KR system (Sympatec, Clausthal, DE). The PM_2.5_ material was dispersed in a 50-mL cuvette under constant stirring at 1200 rpm. The cuvette was filled with a solution of 0.8 g/kg Triton X-100 as a dispersant. A lens with a nominal measurement range from 0.5 to 175 μm was used. Ultra-sonication of 60 s was applied for the material to be properly dispersed. The quantity loaded in the measurement cell cuvette was in the mg range.

The water content was measured on a 758 KFD Titrino volumetric Karl Fischer titrator, (Metrohm, Herisau, CH).

### Electron microscopy

A field emission scanning electron microscope from JEOL JSM7800F (Japan Electron Optics Laboratory Co, Akishima, Tokyo, JP) was used to obtain images of the dust material. Samples were analysed in powder form, without any prior treatment. A small amount of each sample was deposited with a spatula on a double-sided adhesive carbon tape and stuck on an aluminium stub. The analyses were carried out in a qualitative mode, so as to determine the shape of the particles and roughly estimate their size. The presence of the major elements was checked with (energy dispersive x-ray analysis) EDX analysis (AZTEC EDS software, Oxford Instruments, High Wycombe, UK). Details about the magnification (mag.), detector, energy (En.), detection mode and working distance (WD) used for the analysis are reported at the bottom part of each image.

### Inductively coupled plasma atomic emission spectrometry

Na^+^, Ca^2+^, K^+^ and Mg^2+^ were measured using an Agilent 5100 inductively coupled plasma atomic emission spectrometry (ICP-AES) system (Santa Clara, CA, USA) and calibrated with mono element solutions from SpexCertiprep (Metuchen, NJ, USA). The calibration standards were prepared in the interval ranging from 0 to 50 mg/L, depending on the ion, and in all instances evaluated with a linear equation.

### Flow injection analysis

NH_4_^+^ was measured using a SAN flow injection analysis (Skalar, Breda, NL).

## Results and discussion

For the purpose of producing a CRM by a collection of naturally occurring PM_2.5_, an approach was described by Heller-Zeisler et al. using collections on filters by suspending ambient particulate matter in ultra-pure water [13]. This principle was later used to produce NIST SRM 2783. In another paper, the authors used cyclone-based collection on Teflon filters [14]. The latter paper illustrates the major difficulty in obtaining sufficient amounts of material where only 11.5 g was collected over 5 weeks of operation of the cyclone separator. In another paper, Schantz et al. describe a collection of naturally occurring PM_2.5_ over multiple years with a yield of only gram-quantity samples (sic*.*) [15]. A major obstacle for the production of a CRM is not only the lack of access to sufficient amounts of material but also access to material that is similar as possible to authentic air-sampled PM_2.5_. Long periods of the collection also induce considerable heterogeneity in the material as the composition of the particles varies with time, wind direction, weather and seasons. The need for homogenisation of such materials is another technical obstacle that must be tackled, even if enough material is collected. A compromise is, therefore, required regarding access to sufficient amounts of material and the nature and origin of this material. Ideally, the material should be authentic air-sampled PM_2.5_. But for the reasons discussed above, an approximation based on, for example, filtration of suspended fine particles, re-suspension of existing particulate matter materials or jet-milling of roadside dust has been used to produce the already existing air particulate matter CRMs from NIST (SRM2783, SRM2786 and SRM2787) and the EC-JRC (ERM-CZ100 and ERM-CZ120), respectively [10, 11, 13, 14].

**Fig. 1:**
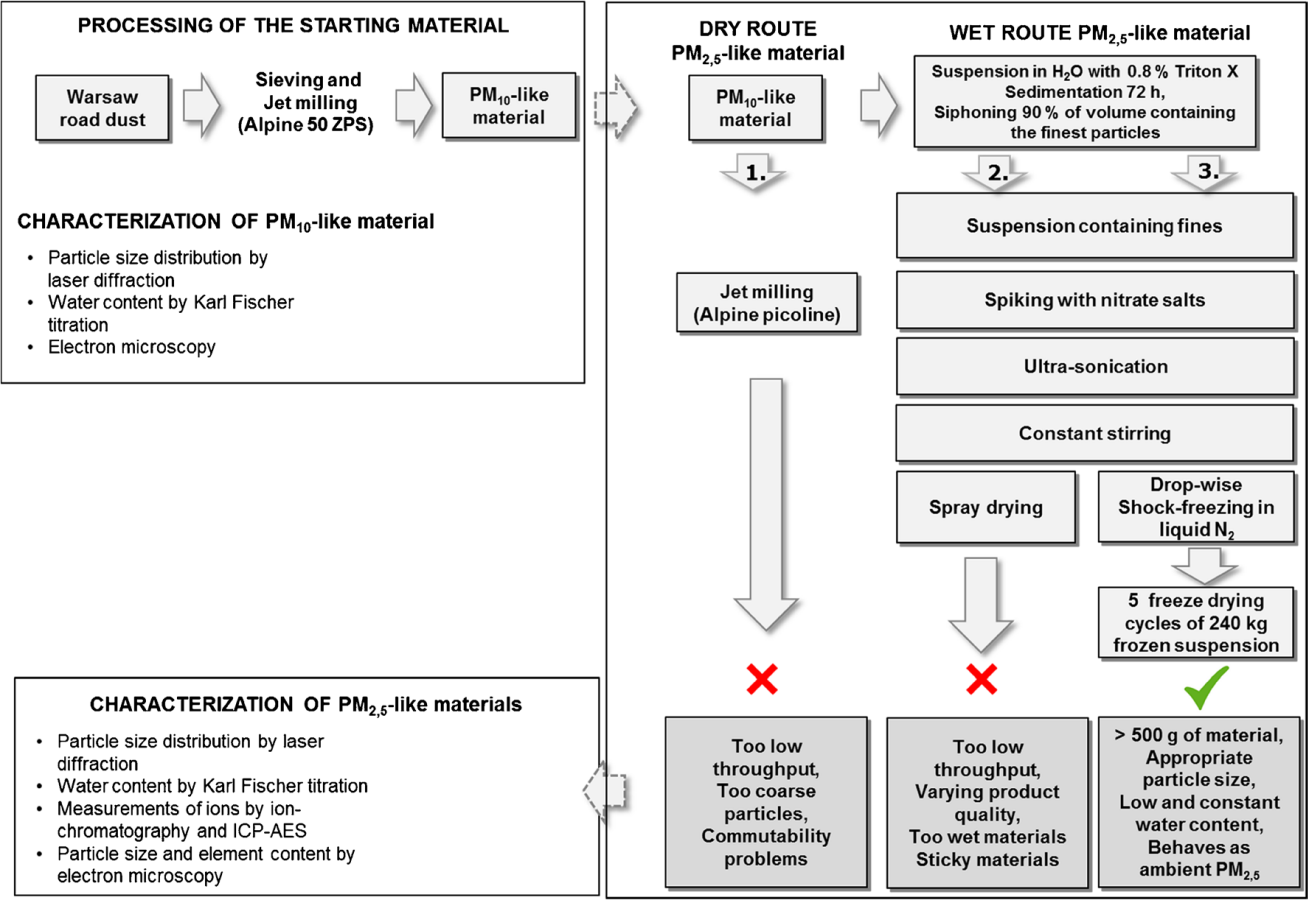
Schematic overview of the three different routes numbered 1, 2 and 3 investigated for the preparation of the PM2.5-like material

As can be seen in Fig. 1, three routes have been explored to produce a sufficient amount of fine dust. In route 1, the PM_10_-like material, obtained from the road tunnel dust, was milled further using another jet mill (Alpine, Picoline). This equipment has the potential to reach top particle sizes well below 10 μm. Despite numerous attempts in balancing air-flow, the speed of the classifier wheel and the speed of infeed of the jet-mill and the vacuum applied on the Teflon-coated collection filter, it was not possible to obtain particles of sufficiently low diameter with the prospect of producing sufficient quantities for a CRM. Also, the resulting particles did not behave in the same way in the analytical process in comparison with dust collected on filters because of much longer extraction times necessary for the complete liberation of the ions.

Secondly, an approach based on spray-drying was attempted following apparently successful initial trials as depicted in route 2 in Fig. 1. First, the coarse particles in the PM_10_-like material were removed by sedimentation. According to the Mason-Weaver equation, particles with larger hydrodynamic diameters sediment at a higher speed than smaller particles [16]. This approach is based on a wet route where some alteration of the chemical composition is inevitable because soluble components are dissolved, as pointed out by Heller-Zeiser et al. [13, 14]. On the other hand, since the solvent (water) is removed during spray drying, the solutes will adsorb back onto the particles during drying in contrast to a method only based on filtration [13]. Also for this approach, numerous attempts were made to optimise the temperature for drying, by changing airflows, temperature, pump-speeds, etc. Unfortunately, the resulting powder was of variable product quality, sometimes too wet and sticky, hence also making homogenisation next to impossible. Also, the yield was too low with the prospect of producing enough bulk material for a CRM production. Moreover, it can further be argued that nowhere in the atmosphere are there conditions of > 200 °C (conditions inside the spray-dryer), which makes this approach the least appropriate of the three investigated routes. Because of the technical problems and poor quality of the material, this approach was also abandoned.

The third approach was based on freeze-drying, following shock-freezing of the suspension in liquid nitrogen as shown in route 3 in Fig. 1. This approach is indeed more similar to natural processes where ice formation around nuclei in the air masses can take place also in warmer climates whereby particles collected directly on filters may have been subjected to freezing/thawing of particle–water interfaces. Also here, dissolved species are re-absorbed onto the particles in contrast to filtered PM-materials [13]. Given the results of appropriate particle size and good commutability, this approach was the most suitable method for the subsequent preparation of a sufficient amount of PM_2.5_ like material. The use of 0.8% Triton X in the suspension was found necessary for dispersion of the particulate matter. Fortunately, the presence of Triton-X after freeze drying does not induce foaming or other undesirable effects when the dust is re-suspended and extracted prior to analysis by IC. Consequently, all the data presented in continuation is only concerned with the final successful approach based on shock-freezing and freeze drying.

### Particle size—approaching the EN12341 definition of PM_10_ and PM_2.5_

**Fig. 2:**
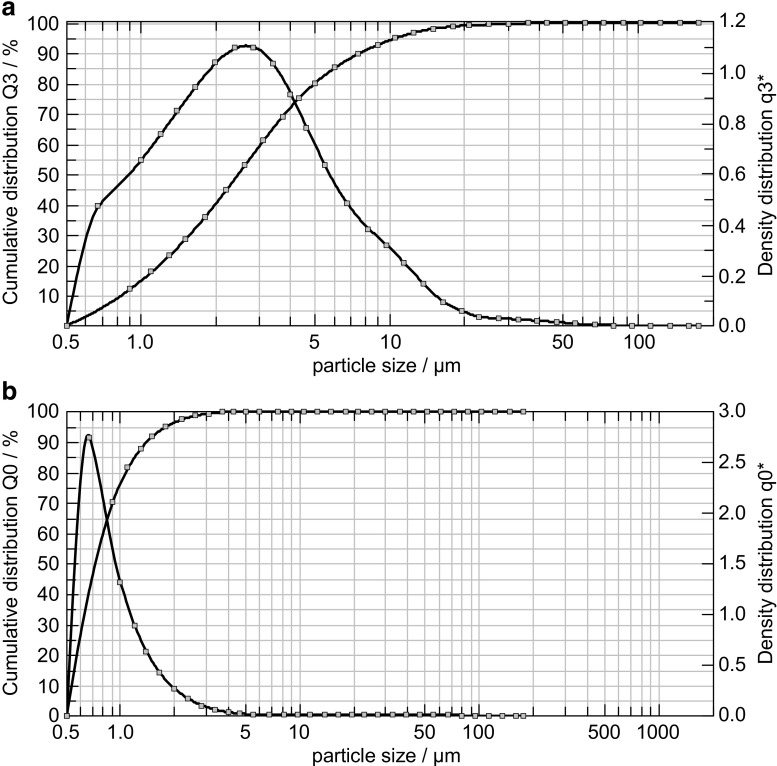
**a** Particle volume size distribution (Q3) of the PM2.5-like material where X50 is 2.47 ± 0.17 μm. The fine dust was dispersed in isopropanol and measured after 30-s ultrasonication. The curves displayed here are the average of data from five freeze-drying batches. **b** Particle number distribution (Q0) of the PM2.5-like material where X50 is 0.79 μm and X95 is 1.92 μm. The fine dust was dispersed in isopropanol and measured after 30-s ultrasonication

**Fig. 3:**
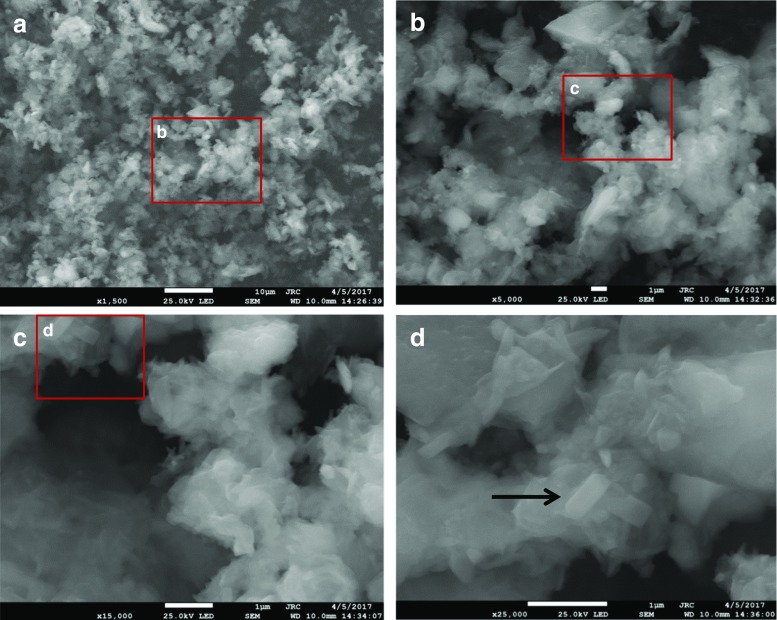
Electron microscopy images of the PM2.5-like material. Red lined squares indicate the area presented in the subsequent image in alphabetical order. **a** (×1500 magnification) Followed by the full area in (**b**) (×5000) followed by (**c**) (×15,000) and (**d**) of ×25,000 magnification. The white horizontal bar at the bottom of each image is indicating the length scale, i.e. either 10 μm (**a**) or 1 μm (**b**–**d**)

As can be seen in Fig. 2a, b, the measurements based on laser diffraction indicate that the particles in all batches approach the EN12341 definition of PM_2.5_ particles since 50% of the cumulative *volume* distribution has particle size diameters below 2.5 μm, and 95% of the particles in the *number* distribution is below 2 μm. To test if the particles fulfil the EN12341 definition, it would be necessary to re-suspend a known amount of this reference material in air and sample it using a standard sampler to check if a 50% sampling efficiency can be reached. In practice, this would be rather difficult to achieve, especially to produce and control concentrations at μg/m^3^ level in a defined volume of air. Also, as shown in Fig. 3a–d, the images obtained using electron microscopy confirm the presence of particles mainly below 2.5 μm of particle size. Such particles are small enough to remain suspended in the air for longer periods of time due to their low settling speed in calm air [17]. One must realise that the aerodynamic diameter is different from the physical diameter even when considering a spherical particle because of the definition. As could be expected, the images (Fig. 3a–d) show particles that are irregular in shape. The aerodynamic diameter of the rectangular particle marked with an arrow in Fig. 3d is consequently defined as the diameter of a hypothetical sphere of density 1 g/cm^3,^ having the same terminal settling velocity in calm air as the particle in question (regardless of its geometric size, shape and true density) [4]. The electron microscopy also provided a crude elemental composition of the particles when operated in the EDX mode as reported in Table 2. Although the values can only be considered as being indicative, it, however, shows the presence of elements such as oxygen, silicon, calcium, sulphur, sodium and chloride in falling order, which could correspond to the presence of silicon oxide, calcium oxide and sulphates, respectively. As was confirmed in the EDX data, the elements added by spiking were also found, although the methods for characterisation will mainly be based on ion chromatography and inductively coupled plasma atomic emission.

**Table 2:** Summary of EDX analyses on the PM_2,5_-like material from the five freeze-drying cycles (the amount of each element in mass % should be considered as indicative only)

Element (mass %)	Batch 1	Batch 2	Batch 3	Batch 4	Batch 5
O	50	54	49	52	57
Na	6.2	6.6	5.4	6.6	6.7
Mg	1.8	1.7	1.7	1.6	1.5
Al	4.0	3.7	4.1	3.8	3.6
Si	12	12	13	11	10
P	–	0.43	–	–	0.37
S	6.2	5.8	5.8	6.6	5.9
Cl	4.5	3.7	4.9	4.9	3.7
K	1.3	0.9	1.7	1.4	1.1
Ca	9.3	7.9	10.3	9.0	6.8
Ti	0.41	0.28	0.36	0.31	0.30
Fe	3.5	2.9	3.6	2.8	2.5
Cu	0.36	0.33	0.21	0.25	0.46
Zn	–	–	0.20	–	–

#### Water content—hygroscopicity and handling of the material

**Fig. 4:**
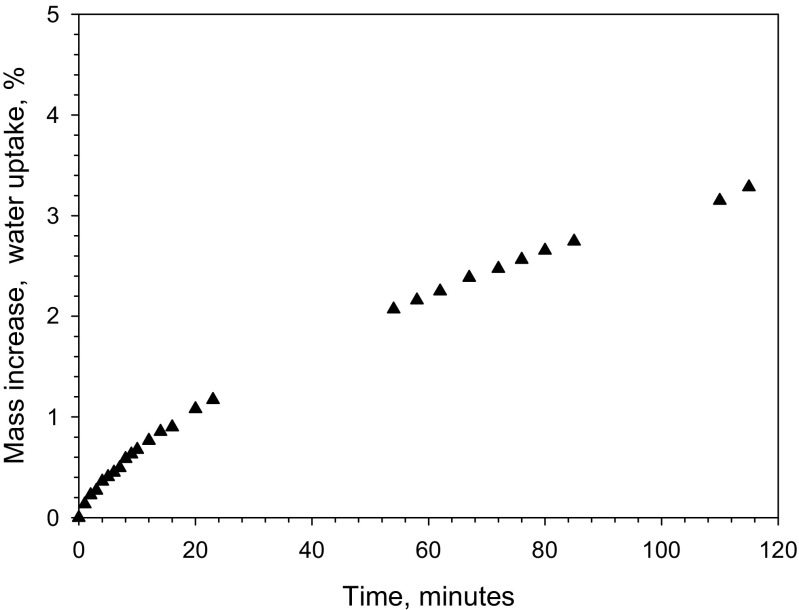
Water uptake on the PM2.5-like material as a function of time at 70% RH

Certified values are commonly reported based on dry mass in matrix reference materials. This requires a correct measurement of water content, but inevitably increases the uncertainty of measurement, especially if the method for determining water is imprecise. Also, a separate portion of the reference material must be used to determine water content. If only small amounts of the CRM are available, in this case, 150 mg per vial, the need for extra material for establishing water content constitutes an additional impediment. To this end, the material produced here will be certified without correction for its water content such that users can use it as supplied. Measurements of water content using volumetric Karl Fischer titration showed that the water content was around 4.5% water (m/m). Material from five different freeze-drying cycles was measured in four vials per batch, as reported in Table 3. Only one replicate per vial was possible because of the limited amount of material per unit. As shown in Fig. 4, the maximum handling period in a typical lab environment is about 15 min to maintain the pick-up of water below 1% relative to the mass. The absorption curve was obtained at an ambient humidity of 70% RH. Consequently, the powder is slightly hygroscopic, but can still be handled and weighed in a normal lab environment. It is advisable to store opened units in a desiccator to prevent the degradation of the material.

**Table 3:** Water content in the PM_2,5_-like reference material measured by volumetric Karl Fischer titration. Four units were measured per freeze drying batch with one replicate per unit

Freeze-drying batch	Water content ± 1SD, % (m/m)
First batch	4.88 ± 0.41
Second batch	4.77 ± 0.29
Third batch	4.66 ± 0.16
Fourth batch	4.41 ± 0.32
Fifth batch	4.42 ± 0.38
All batches	4.63 ± 0.21

#### Extractability of ions—comparison between the PM_2.5_-like material and authentic air-sampled PM_2.5_ on filters

**Fig. 5:**
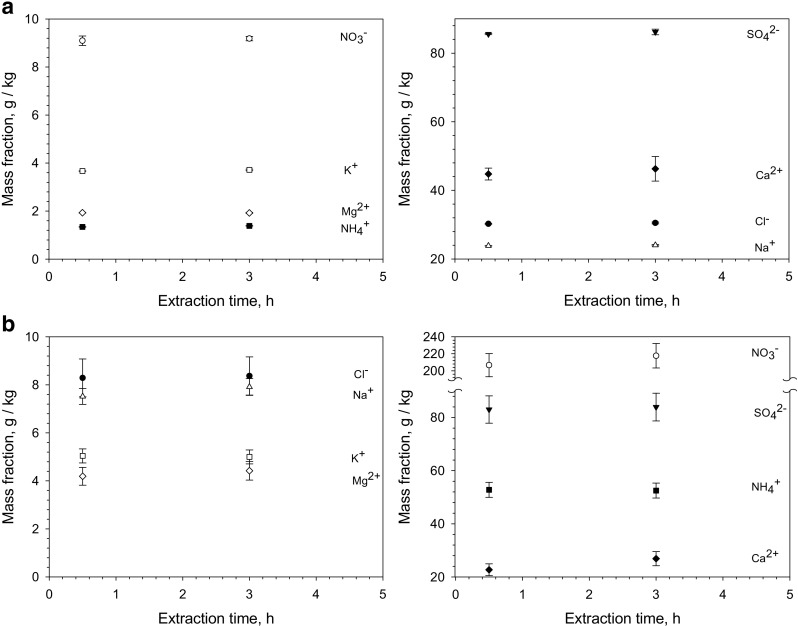
**a** Extractability of ions from PM_2.5_-like material obtained by freeze drying a suspension with fine particles. **b** Extractability of ions from authentic air-sampled PM_2.5_ material from the Antwerp region in Belgium

When a reference material and a routine sample behave in the same way in the analytical process, they are commutable [18]. This property is a fundamental criterion to consider for any reference material. In this case, it must be demonstrated that the ions present in the synthetically produced PM_2.5_-like material behave in the same way during the extraction process as the ions present in authentic air-sampled PM_2.5_. According to the CEN/TR 16269:2011 method for analysis by ion chromatography, the extraction time was set to 30 min for exhaustive extraction of ions on filters. In Fig. 5a (PM_2.5_-like material) and Fig. 5b (authentic air-sampled PM_2.5_ on filters), evidence is presented for the extraction yield of the eight ions after 0.5 and 3 h ultra-sonication, respectively. As can be seen, there is no increase in the amount of ions extracted from any of the materials by increasing the extraction time to 3 h. The error bars displayed in Fig. 5a are ± two standard deviations based on three replicate analyses using 25 mg of PM_2.5_-like material. In Fig. 5b, the error bars are ± one standard deviation of four replicates analysing three filters cut in four equal parts. The variation in the results is higher in Fig. 5b because of the local heterogeneity on the filter surface in contrast to the highly homogeneous powder that is obtained after freeze drying a suspension. The results clearly show that the synthetic PM_2.5_-like material behaves like air-sampled PM_2.5_ material with respect to the extraction of ions. Further aspects of commutability related to synthetic PM_2.5_-like materials and authentic air-sampled PM_2.5_ material are explained and discussed in a separate paper that is currently under preparation by Emma et al. [19].

#### Putting together data from different freeze-drying batches—assessment of between bottle heterogeneity using ANOVA

At the onset, the batch from the first freeze-drying cycle was removed from further evaluation as the levels of the ions to be certified were substantially lower in comparison with the remaining four freeze-drying batches. Currently, no explanation can be given for this discrepancy. The between-bottle heterogeneity (u_bb_) was, after that, investigated by evaluating measurement data of the target parameters from the remaining four freeze-drying cycles. The data for Na^+^, Mg^2+^ K^+^ and Ca^2+^ was generated using ICP-AES while ion chromatography was used to measure Cl^−^, NO_3_^−^ and SO_4_^2−^. For NH_4_^+^, a Skalar, SAN flow injection analysis system was used as mentioned above. The evaluation of homogeneity for all ions was based on the analysis of variance (ANOVA) using three replicate measurements in each sample unit according to ISO Guide 35 [20]. In this way, a value in percent can be obtained that corresponds to the between-bottle heterogeneity of each analyte. This contribution of between-bottle heterogeneity will make up a part of the final uncertainty at the time of assigning the certified values and their uncertainties. Freeze-drying batch 2–5 finally made up 2161 filled units, followed by freeze-drying batch 2–4 which corresponded to 1786 units and finally freeze-drying batch 3–5, comprised 1575 units. In each freeze-drying batch, five different sample units were analysed; in total, between 15 and 20 units were used for the homogeneity testing depending on the number of batches investigated. Also, analytical data corrected for moisture content in the material was similarly investigated. The results were then evaluated on the significance to trends in fill-order and analytical sequence at 95 and 99% significance, respectively. In case a trend in fill-order was detected, the uncertainty component for between-bottle heterogeneity (u_bb_) was obtained using the rectangular distribution [20]. No outlying values were excluded in the calculations, and the data distribution was checked for uni-modality and normal distribution. In Tables 4 and 5, all the data is compiled while the data in Table 6 shows the between-bottle heterogeneity of the eight ions to be certified when using batch 2–5 without applying the dry mass correction. As can be seen in Table 6, the u_bb_ varies between 2 and 5% for all ions except ammonium which has a u_bb_ of 8.5%. Consequently, it would be feasible to certify seven out of eight ions in this material, based on the homogeneity data, whereas for ammonium, one could consider an indicative value. This reference material is currently undergoing stability studies. Characterisation measurements are also under way. All steps will be performed under ISO Guide 34 accreditation applying procedures described in ISO Guide 35 [12, 20].

**Table 4:** Evaluation of trends in fill order, analytical sequence and calculation of u_bb_ based on different freeze-drying batches for the PM_2.5_-like material *without* correction for water content; data in bold to be used for the u_bb_ component

Analyte	Batch 2–5/2161 units						Batch 2–4/1786 units						Batch 3–5/1575 units					
	Trend fill order 95/99		Analytical trend 95/99		u_bb_/%	u_rec_	Trend fill order 95/99		Analytical trend 95/99		u_bb_/%	u_rec_	Trend fill order 95/99		Analytical trend 95/99		u_bb_/%	u_rec_
Na^+^	–	–	Y	Y	**1**.**7**		–	–	–	–	**2**.**2**		–	–	Y	Y	**1**.**0**	
Ca^2+^	–	–	–	–	**3**.**6**		–	–	–	–	**3**.**1**		–	–	–	–	**4**.**2**	
K^+^	Y	Y	Y	Y	2.6	**4**.**0**	Y	Y	–	–	3.0	**3**.**9**	–	–	Y		1.4	**4**.**3**
Mg^2+^	Y	Y	Y	–	5.5	**5**.**1**	Y	Y	–	–	4.4	**5**.**1**	Y	Y	Y	–	**3**.**6**	
Cl^−^	Y	–	Y	Y	1.8	**1**.**9**	Y	Y	–	–	1.7	**1**.**9**	–	–	Y	–	**1**.**7**	
NO_3_^−^	Y	Y	Y	–	2.0	**2**.**2**	Y	Y	–	–	1.8	**2**.**2**	–	–	–	–	**1**.**6**	
SO_4_^2−^	–	–	Y	–	**4**.**3**		–	–	–	–	**3**.**5**		–	–	–	–	**5**.**5**	
NH_4_^+^	Y	Y	–	–	13.9	**8**.**5**	Y	–	–	–	9.8	**8**.**5**	Y	–	–	–	9.8	**9**.**1**

**Table 5:** Evaluation of trends in fill order, analytical sequence and calculation of u_bb_ based on different freeze-drying batches for the PM_2,5_-like material *with* correction for water content, data in bold to be used for the u_bb_ component

Analyte	Batch 2–5/2161 units						Batch 2–4/1786 units						Batch 3–5/1575 units					
	Trend fill order 95/99	Analytical trend 95/99			u_bb_/%	u_rec_	Trend fill order 95/99		Analytical trend 95/99		u_bb_/%	u_rec_	Trend fill order 95/99		Analytical trend 95/99		u_bb_/%	u_rec_
Na^+^	–	–	Y	Y	**1**.**8**		–	–	–	–	**2**.**1**		–	–	Y	Y	**1**.**2**	
Ca^2+^	–	–	–	–	**3**.**5**		–	–	–	–	**2**.**6**		–	–	–	–	**4**.**2**	
K^+^	Y	Y	Y	Y	2.4	**3**.**7**	Y	Y	–	–	2.9	**3**.**7**	–	–	Y	Y	**4**.**2**	
Mg^2+^	Y	Y	Y	–	5.2	**5**.**1**	Y	Y	–	–	4.5	**5**.**1**	Y	–	Y	–	3.3	**4**.**1**
Cl^−^	–	–	Y	Y	1.7	**1**.**9**	Y	Y	–	–	1.5	**1**.**9**	–	–	Y	–	**1**.**8**	
NO_3_^−^	Y	–	Y	–	1.7	**2**.**0**	Y	Y	–	–	1.6	**2**.**0**	–	–	–	–	**1**.**5**	
SO_4_^2−^	–	–	Y	–	**4**.**3**		–	–	–	–	**3**.**3**		–	–	–	–	**5**.**6**	
NH_4_^+^	Y	Y	–	–	13.7	**8**.**5**	Y	–	–	–	9.9	**8**.**5**	–	–	–	–	**9**.**5**

**Table 6:** Maximum u_bb_ in % using freeze-drying batches 2–5 without correction for moisture content

Analyte	Batch 2–5, u_bb_/%
Na^+^	1.7
Ca^2+^	3.6
K^+^	4.0
Mg^2+^	5.1
Cl^−^	1.9
NO_3_^−^	2.2
SO_4_^2−^	4.3
NH_4_^+^	8.5

## Conclusions

A substantial amount of a sufficiently homogeneous PM_2.5_-like reference material has been produced from ambient dust and filled in vials. It will be used for quality assurance of measurements of ions common to fine atmospheric dust of natural origin. Despite many technical and physico-chemical challenges, the reference material has been proved to be sufficiently homogeneous and it has been shown that the ions in the PM_2.5_-like material behave in the same way in the analytical process as ions in air-sampled PM_2.5_ dust on filters. In this respect, the two material types are interchangeable. The future certified reference material will support the implementation of European Directive 2008/50/EC. Future developments using this kind of material preparations can be envisaged if for example, polycyclic aromatic hydrocarbons, trace elements or other compounds of interest have to be present in a PM_2.5_ matrix. Since the sample preparation route involves a suspension, spiking and homogenising such analytes with the PM-material should be relatively straightforward. It is not expected that major losses of analyte would take place during shock-freezing of this suspension or during the subsequent freeze-drying step.
